# From Summer Facilitation to Winter Avoidance: Seasonal Shifts in Livestock‐Wild Ungulate Temporal Coexistence

**DOI:** 10.1002/ece3.73705

**Published:** 2026-05-24

**Authors:** Melissa C. B. Einsele, Sandeep Sharma, Matthias Waltert, Henrique M. Pereira, Annika M. Zuleger

**Affiliations:** ^1^ Department of Conservation Biology Georg‐August Universität Göttingen Göttingen Germany; ^2^ Lund University Lund Sweden; ^3^ Institute of Biology Martin‐Luther‐Universität Halle (Wittenberg) Halle (Saale) Germany; ^4^ German Centre for Integrative Biodiversity Research Leipzig Germany; ^5^ IUCN SSC Bear Specialist Group Washington DC USA; ^6^ Leibniz Institute for the Analysis of Biodiversity Change, Museum Koenig Bonn Bonn Germany; ^7^ BIOPOLIS Program in Genomics, Biodiversity and Land Planning CIBIO, Campus de Vairão Vairão Portugal

**Keywords:** activity patterns, agricultural rewilding, coexistence, herbivores, livestock, seasonality

## Abstract

Traditional pastoralism has long shaped Southern European ecosystems, but widespread agricultural abandonment is transforming these landscapes. Yet, extensive livestock grazing remains a persistent driver of ecological dynamics. Simultaneously, the concept of agricultural rewilding has gained attention as a means of using domestic livestock to restore functions once filled by wild ungulates. However, the ecological interactions between livestock and native wild ungulates, especially across seasons, remain poorly understood. Using long‐term camera trap data from Peneda‐Gerês National Park, Portugal, we examined how free‐ranging cattle and horses influence the spatiotemporal activity of roe deer and wild boar across summer and winter. We assessed diel activity levels and patterns and applied generalized linear mixed models to evaluate the effects of livestock abundance and activity, as well as the presence of herding dogs, using encounter rates as a proxy for livestock abundance. Cattle and horses were predominantly diurnal, whereas roe deer and wild boar were mainly crepuscular and nocturnal, showing increased nocturnality in summer. Both wild ungulate species showed seasonal differences in response to cattle: activity was higher at high cattle abundance sites in summer but declined in winter. This pattern suggests that grazing may facilitate resource access when plant productivity is high but drive temporal avoidance and competition when resources are limited. Herding dog presence further modified these responses, reinforcing positive associations in summer, particularly outside of core cattle activity periods. In contrast, horse presence was associated with higher wild ungulate activity across seasons and times of day, indicating facilitative interactions. This highlights the seasonal variability in wildlife‐livestock relationships and demonstrates that conclusions based solely on summer data risk underestimating livestock impacts. It further underscores the need for year‐round monitoring and adaptive, species‐specific grazing strategies that account for livestock‐associated disturbance factors such as herding dogs to balance biodiversity restoration with sustainable pastoral management.

## Graphical abstract

Agricultural abandonment in Southern Europe has made domestic livestock central ecological drivers, yet their interactions with wild ungulates remain poorly understood. Using long‐term camera trap data from northern Portugal, we assessed spatiotemporal effects of livestock on wildlife and found strong seasonal contrasts: cattle facilitated roe deer and wild boar activity in summer but reduced activity in winter, whereas feral horses were consistently associated with higher wild ungulate activity. These findings highlight pronounced seasonal variability in wildlife–livestock interactions and the importance of year‐round monitoring.

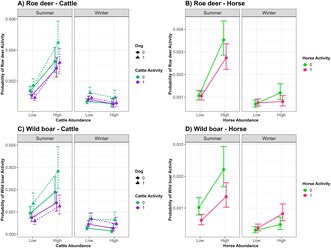

## 1 Introduction

Many European landscapes have a long history of human land use, where agriculture and pastoralism over thousands of years have shaped ecosystems, species distributions, and biodiversity (Ellis 2021). However, in recent decades, large‐scale human exodus from rural areas has led to widespread abandonment of agricultural land, particularly in southern Europe, where migration to cities has caused significant population declines (Lasanta et al. 2017; Pereira and Navarro 2015). While this can lead to the restoration of native vegetation, recolonization of native species, and the creation of more heterogeneous habitats (Boitani and Linnell 2015; Navarro and Pereira 2012), it can also increase human‐wildlife conflicts. In many of these regions, extensive livestock grazing remains one of the few persistent land‐use practices that continues to influence ecological processes and species interactions (Martin‐Díaz et al. 2020).

At the same time, the European Union's Biodiversity Strategy for 2030, which aims to protect at least 30% of EU land and sea, has renewed interest in agricultural rewilding or “Rewilding Lite” approaches (Corson et al. 2022; Gordon, Pérez‐Barbería, and Manning 2021; Rey Benayas et al. 2025). Unlike holistic rewilding, which relies on the reintroduction of missing wild herbivores and predators (Perino et al. 2019; Svenning et al. 2016), agricultural rewilding integrates domestic livestock into conservation management to restore ecological functions in human‐modified landscapes (Corson et al. 2022; Gordon, Manning, et al. 2021; Gordon, Pérez‐Barbería, and Manning 2021). By using locally adapted or semi‐feral livestock breeds to restore the grazing and browsing roles of extinct or locally lost megafauna, this approach seeks to combine biodiversity restoration with continued agricultural use and rural livelihoods (Gordon, Pérez‐Barbería, and Manning 2021).

This type of managed grazing can play a valuable ecological role by maintaining habitat diversity, limiting shrub encroachment, and partially replicating the ecological functions of native herbivores, for example by creating or maintaining open foraging areas and improving forage quality, a process often described as *grazing facilitation* (Gordon, Pérez‐Barbería, and Manning 2021; Hall 2018; Vavra 2005). Yet, livestock and wild ungulates may also engage in negative interactions, including competition, disturbance, habitat modification, reduced environmental quality, and potential disease transmission (Putman et al. 2011; Triguero‐Ocaña et al. 2019). In areas with high resource and spatial overlap, livestock may significantly impact wild ungulates by increasing physiological and ecological stress, altering their spatial and temporal behavior, and displacing them from preferred habitats (Carter et al. 2014; Chaikina and Ruckstuhl 2006; Feng et al. 2021; Mori et al. 2020; Putman and Apollonio 2014; Putman 1996; Rebollo et al. 1993; Triguero‐Ocaña et al. 2019; Zhang et al. 2017). Beyond these behavioral and ecological effects, shared use of space may also influence pathogen transmission dynamics at the livestock–wildlife interface, linking disturbance processes to broader animal and public health considerations within a One Health framework (Zinsstag et al. 2011; Wiethoelter et al. 2015; ENETWILD‐Consortium 2021).

Studies on coexisting wild and domestic ungulates show mixed outcomes, ranging from facilitation to competition, with low livestock densities often benefiting wild ungulates and high densities having negative effects (Barroso and Gortázar 2024; Filazzola et al. 2020; Herfindal et al. 2017; Roberts et al. 2021). These mixed responses, combined with challenges in detecting and quantifying interactions, complicate predictions for the ecological implications of extensive farming systems or livestock‐based rewilding and the design of management strategies at the wildlife‐livestock interface (Triguero‐Ocaña et al. 2019). While agricultural rewilding research has focused largely on social and management dimensions, its ecological effects on native species and habitats remain less explored (e.g., Van Dooren et al. 2025). Yet, understanding how livestock influence wild ungulates, particularly through spatial and temporal overlap, is essential for assessing the sustainability and scalability of rewilding and low‐intensity pastoralism in abandoned landscapes (Caravaggi et al. 2017).

Spatiotemporal activity patterns are shaped by biotic and abiotic factors, including competition, habitat structure, and temperature (Herfindal et al. 2017; Kukielka et al. 2013; Triguero‐Ocaña et al. 2019), with seasonal changes in resources, photoperiod, climate, and husbandry further modifying behavior (Triguero‐Ocaña et al. 2019; Wang et al. 2024). Many species alter activity to avoid thermal stress, reducing heat exposure in summer (Maloney et al. 2005) and cold in winter (Du Toit and Yetman 2005), resulting in more crepuscular or nocturnal patterns during hot periods (Scheibe et al. 2009) and increased diurnal overlap with livestock in winter (Herfindal et al. 2017; Triguero‐Ocaña et al. 2019). While many studies originate from subtropical systems such as South Africa (e.g., Maloney et al. 2005; Du Toit and Yetman 2005), the thermoregulatory mechanisms involved are broadly applicable across climatic regions, and similar responses have been documented in temperate systems with strong seasonal contrasts (Herfindal et al. 2017; Triguero‐Ocaña et al. 2019). Habitat structure further mediates interactions: for example, in Doñana, Spain, cattle‐wild boar encounters were most frequent near water and lowest in dense vegetation (Triguero‐Ocaña et al. 2019), while in South Central Spain, water points became hotspots for indirect interactions during dry periods (Kukielka et al. 2013).

Additionally, behavioral responses to disturbance shape distributions, with wildlife often shifting toward nocturnality to avoid humans and livestock (Gaynor et al. 2018). In Europe, several studies have shown an increase in nocturnal activity in response to human disturbance such as recreation and tourism, hunting, forestry, and agricultural activities (e.g., Drimaj et al. 2021; Torretta et al. 2025; Johann et al. 2020; Wevers et al. 2020). Evidence for livestock‐mediated disturbance within Europe, although comparatively limited, suggests similar ecological consequences. Roe deer in Spain exhibited elevated stress indicators (fecal cortisol metabolites) in areas shared with cattle, indicating physiological costs associated with livestock presence (Horcajada‐Sánchez et al. 2019). In Mediterranean woodland systems, livestock grazing alters vegetation structure and forage availability, thereby influencing habitat use and resource distribution for wild ungulates (Acevedo et al. 2007). At a broader scale, extensive spatial overlap between livestock and wild ungulates across Europe highlights the widespread potential for interaction and disturbance at the livestock–wildlife interface (ENETWILD‐Consortium 2021).

Outside Europe, behavioral responses to livestock are more extensively reported. In northern China, cattle reduced diurnal activity of wild boar and sika deer (Feng et al. 2021), while in the Serengeti, wild herbivores became more crepuscular in areas grazed by cattle (Guthmann et al. 2024). However, avoidance is not exclusively temporal: in the Indian trans‐Himalaya, wild ungulates primarily avoided livestock through fine‐scale spatio‐temporal segregation (Bhasin et al. 2024), while in Mongolia, higher cattle abundance reduced wapiti numbers, whereas horses increased wapiti and roe deer occurrence (Mazzamuto et al. 2025). Conversely, in North America, resource partitioning between horses and native ungulates led to spatial and temporal avoidance of shared water sources (Hall et al. 2018). Although such spatiotemporal shifts may reduce direct contact, they can constrain foraging and increase predation risk (Guthmann et al. 2024; Mori et al. 2020; Sih et al. 2000). In addition, domestic herding dogs may amplify disturbance effects. They can disrupt normal activities such as movement and foraging (Hansen and Smith 1999), and in some regions free‐ranging dogs have been reported attacking native species (Home et al. 2018). Consequently, the combined presence of livestock, humans, and associated dogs may represent both a disturbance and a predation risk, reinforcing avoidance behavior in wild ungulates (Bhasin et al. 2024). Despite growing interest in wildlife‐livestock interactions, knowledge of their spatiotemporal dynamics in European landscapes remains limited (Horcajada‐Sánchez et al. 2019; Mori et al. 2020). Research has traditionally focused on spatial distribution patterns, using approaches such as occupancy or co‐occupancy models, but this emphasis may overlook important temporal dimensions of species interactions and their impacts on wild ungulates (Frey et al. 2017; Wilson et al. 2020). Recent studies have begun to explore the temporal aspects of habitat use and activity, yet many remain constrained in scope, often restricted to summer months, lacking seasonal differentiation, or conducted in regions with minimal seasonal variation (Franchetto et al. 2025; Gaudiano et al. 2021; Guthmann et al. 2024; Mazzamuto et al. 2025). Consequently, our understanding of how wild ungulates respond to livestock presence across periods of low resource availability and how this influences temporal overlap and coexistence remains incomplete.

To address this knowledge gap, we examine the impact of domestic livestock on the spatiotemporal distribution of wild ungulates. Specifically, we test (1) whether free‐ranging cattle and semi‐feral horses, as well as the presence of herding dogs, influence the spatial and temporal dynamics of roe deer and wild boar, and (2) whether these interactions differ between summer and winter. We use long‐term camera trap data from the Peneda‐Gerês National Park in northern Portugal, a region with substantial population declines and agricultural abandonment, where extensive cattle husbandry persists and feral horses remain from former grazing systems. Previous work in this area showed broad spatial overlap between livestock and wild ungulates but focused only on spring and summer (Zuleger et al. 2025). Here, we use year‐round data to assess how livestock shapes ungulate spatiotemporal distributions across seasons within a traditional landscape exhibiting rewilding‐like conditions.

## 2 Methods

### 2.1 Study Area

**FIGURE 1:**
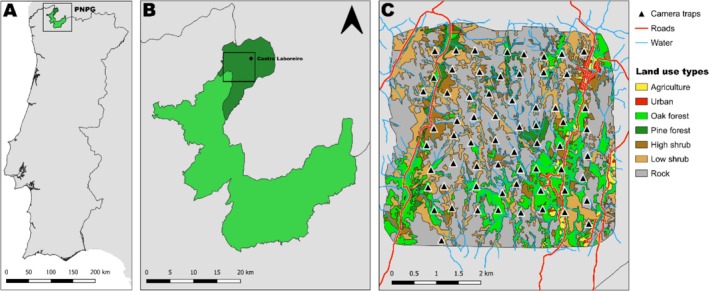
(A, B) Study location in the Peneda‐Gerês National Park (PNPG) in northern Portugal. (C) Camera‐trap locations and land‐use types within the survey area. Cameras were placed randomly with regard to animal density and activity, but the locations were chosen in a way to represent the different land‐use types in the area relative to their overall occurrence (Zuleger et al. 2023).

The study was conducted in the parish of *Castro Laboreiro e Lamas de Mouro*, located within the Peneda‐Gerês National Park (PNPG) in northern Portugal (Figure 1). The national park was established in 1971 and covers an area of almost 700 km^2^. Over the past six decades, this region has seen substantial land‐use change due to the abandonment of traditional agriculture, particularly in Castro Laboreiro, where agropastoral activities were once widespread (van der Zanden et al. 2018). However, socioeconomic transformations led to a sharp population decline beginning in the late 1950s (Rodrigues 2010). Livestock systems in PNPG have long‐standing historical importance, with goats and sheep presenting the main livestock. However, in recent years livestock systems transitioned more toward cattle husbandry, with a reduction in goats and sheep. The park supports two endangered local cattle breeds, Cachena and Barrosã, which make up over half of the bovine population and are predominantly raised for meat production. Most cattle are free‐ranging, with no fencing restricting their movements, allowing them to roam across the entire study area. They are often accompanied by large livestock guardian dogs of the local Castro Laboreiro breed, receive winter feed supplementation, and occur at low livestock densities at approximately 0.2 livestock units (LU) per hectare, well below the EU average of 0.7–0.9 LU/ha (Instituto Nacional de Estatística 2019; Zuleger et al. 2023). In addition, the area is home to Garrano ponies, which roam freely across much of the park with minimal husbandry and management intervention. Breeding is no longer actively regulated, and the population persists in a near‐feral state, now primarily valued for their cultural and touristic significance (Bárcena 2020). The main wild ungulate species present in the study area are European roe deer ( *Capreolus capreolus* Linnaeus, 1758) and wild boar ( *Sus scrofa* Linnaeus, 1758), occurring at densities of approximately 6 individuals/km^2^ and 2.5 individuals/km^2^, respectively (Zuleger et al. 2023), and showing stable occupancy trends (Zuleger et al. 2025). Additionally, the Iberian ibex ( *Capra pyrenaica* Schinz, 1838) is currently repopulating the area following its extinction in Portugal in 1890 (Moço et al. 2006) with its distribution in the study area steadily increasing since 2015 (Zuleger et al. 2025). Moreover, the study area supports a stable population of the Iberian gray wolf (*Canis lupus signatus* Cabrera, 1907). However, wolf abundance in the region is relatively low and predation on wild ungulates is limited, as wolves primarily prey on domestic livestock (Llaneza and López‐Bao 2015). Hunting within the national park is prohibited for all large mammal species except wild boar, for which regulated population control is permitted theoretically year‐round although hunting occurs mostly from late August to February. Nevertheless, poaching of roe deer, Iberian ibex, and wolves remains an ongoing issue.

The study area ranges in elevation from 300 to 1340 m and lies between the Mediterranean and Atlantic biogeographic zones, resulting in a temperate Mediterranean climate (van der Zanden et al. 2018). Between 2015 and 2023, the mean annual temperature was 11.6°C (range: −7.2°C to 37.0°C; own data), with summer (April–September) and winter (October–March) averages of 15.4°C (−1.6°C to 37.0°C) and 7.0°C (−7.2°C to 23.2°C), respectively. Average annual precipitation from 1985 to 2015 was 1858 mm (SNIRH—Sistema Nacional de Informação de Recursos Hídricos 2022). The landscape is highly heterogeneous, comprising shrublands, small pastures, oak and pine forests, and rocky outcrops, and is largely dominated by open, sparsely vegetated areas (Figure 1). Crop‐farming occurs on the nearby plateau but not directly within the study area. Water availability is strongly seasonal: several small to medium‐sized streams dry out during summer, whereas larger river networks, primarily located in the valleys along the margins of the study area, retain water year‐round (Figure 1). The area is intersected by two roads (Figure 1). Yet, due to its remoteness and low population density, traffic volume is low. On weekends, local visitors from surrounding municipalities travel to the area, leading to a temporary increase in daytime traffic. However, most tourism is concentrated in the villages, and only limited recreational activities, such as hiking, occur within the study area itself.

### 2.2 Camera Trap Data

We analyzed data from a long‐term wildlife monitoring program initiated in 2015, in which 64 camera traps (Reconyx Hyperfire HC600, Holmen, WI, USA) were deployed annually (typically April/May to October) across a 16 km^2^ grid southwest of Castro Laboreiro (Zuleger et al. 2023) and year‐round from 2020 onward. Camera placement was designed to ensure proportional representation of the main land‐use types within the study area. Cameras were installed at an average spacing of 500 m and mounted on trees at a height of 50–100 cm above ground, with height and orientation adjusted to local terrain and vegetation to maximize field of view. Detection distances nevertheless varied among sites due to differences in vegetation structure, ranging from open areas to dense shrublands and forest habitats. Since 2020, the majority of the camera traps have remained operational throughout the entire year. All cameras were programmed to capture three consecutive images upon activation, with no delay between trigger events. Camera traps were maintained semiannually, with SD cards collected and batteries replaced in May and October. Detailed information on the camera trap sampling design is provided in Zuleger et al. (2023).

Camera trap images were processed using the platform Agouti (Casaer et al. 2019, https://www.agouti.eu/), where images were automatically grouped into sequences of continuous images when < 120 s apart. Each sequence represented one distinct observation event. We extracted all observation events of each target species from the camera trap data, recording only species presence or absence. To assess seasonal effects, we pooled all available detections across years into summer (April–September) and winter (October–March). Accordingly, the summer dataset included observations from 2015 to 2023, whereas winter data were primarily available from 2020 to 2023. Because not all camera traps were operational throughout the entire study period (e.g., due to malfunction, theft, or retrieval), the number of active cameras varied among years and seasons. In total, 430 camera deployments contributed to the summer dataset (2015–2023; mean = 48 cameras per year) and 78 deployments to the winter dataset (2020–2023; mean = 26 cameras per year). As our analyses focused on location‐specific effects of livestock on wildlife activity patterns rather than interannual trends or absolute detection frequencies, and accounted for camera identity and sampling effort, variation in sampling intensity among years is unlikely to bias the estimated temporal relationships.

We classified camera trap locations into high and low livestock abundance based on year‐ and season‐specific encounter rates (observations per trap day), which varied considerably among locations despite cattle and horses being common across the area. Higher encounter rates were assumed to indicate greater site use and potential disturbance, and sites were therefore categorized seasonally into high (upper 50%) and low (lower 50%) abundance for cattle and horses separately. In addition, camera locations were classified according to herding dog presence (1 = present, 0 = absent). Although categorizing encounter rates into high and low classes may reduce information and potentially obscure more gradual relationships, this approach facilitates interpretability, avoids imposing a specific functional form on livestock effects, and reduces sensitivity to extreme values in encounter rates.

### 2.3 Data Analysis

#### 2.3.1 Activity Levels and Patterns

Since animals adjust their activity to solar rather than clock time (Nouvellet et al. 2012), we calculated sunrise and sunset times for each date using the *suncalc* package (Thieurmel and Elmarhraoui 2019) in R version 4.5.1 (R Core Team 2025) and converted clock times to solar times using the average anchoring approach of Vazquez et al. (2019), which expresses activity relative to average sunrise and sunset over the study period, weighted by the number of records per date. All following analyses were conducted using solar times.

Temporal activity levels were estimated using nonparametric kernel density estimates (KDE) fitted with the *fitact* function from the R package *activity* (Rowcliffe et al. 2014). The function fits a circular function *f(x)* to the time‐of‐day distribution of detections, and the activity level is estimated from the area under *f(x)*, relative to its maximum (*f*_*max*_). We generated species‐specific KDEs with 100 bootstrap iterations to obtain 95% confidence intervals (Rowcliffe 2022). To compare diurnal and nocturnal activity, we calculated activity within sunrise‐sunset intervals for summer and winter by fitting a full 24‐h model and specifying the radian interval over which to estimate the percentage of time spent active, expressed relative to *f*_*max*_ of the full 24‐h cycle (M. Rowcliffe, pers. comm.).

Differences in activity levels (δ) were tested with *compareAct*, which applies a Wald test to compare two activity level estimates (Rowcliffe et al. 2014), and differences in activity patterns (Δ) were evaluated with *compareCKern*, a randomization test for circular data (Ridout and Linkie 2009; Rowcliffe et al. 2014). This function estimates the overlap coefficient (Δ), which quantifies similarity between two fitted KDE activity functions *f(x)* and *g(x)*, ranging from 0 (no overlap) to 1 (complete overlap). We used the Δ4 coefficient since all sample sizes were > 50 (Ridout and Linkie 2009). Bootstrap resampling with 100 iterations provided standard errors and confidence intervals.

Because of the large sample sizes typical of camera‐trap datasets, small differences in activity metrics can become statistically significant even when activity patterns remain highly overlapping. Therefore, interpretation focused primarily on the magnitude of overlap (Δ) and activity differences (δ), rather than statistical significance alone.

#### 2.3.2 Generalized Linear Mixed Models (GLMMs)

To assess whether ungulate activity differed not only with livestock activity but also livestock relative abundance, we employed binomial generalized linear mixed models (GLMMs) following Guthmann et al. (2024), using camera location as a random effect to account for repeated measurements and unobserved heterogeneity among camera sites (Iannarilli et al. 2021). For each location and season, we created a dataset of 24 1‐h intervals, recording presence or absence of each species by summing the number of days in which at least one detection occurred in a given interval (presence). Absence was calculated as sampling days minus presence. The response variable combined presence and absence to account for variation in sampling effort.

We tested livestock abundance (high/low), livestock activity (0/1), and season (summer/winter) as binary predictors and included their interactions using the R package *lme4* (Bates et al. 2015). In models evaluating cattle effects, we additionally included dog presence (0/1) as a predictor because dogs were associated with cattle herds but not with horses. Core activity periods for cattle and horses were defined from the activity patterns as the hours of maximum activity (cattle: 07:00–22:00 in summer, 09:00–20:00 in winter; horses: 06:00–21:00 in summer, 08:00–22:00 in winter).

Model assumptions were evaluated using simulation‐based diagnostics implemented in the *DHARMa* package (Hartig 2024). Residual diagnostics included Q–Q plots and formal tests for residual uniformity (Kolmogorov–Smirnov test), dispersion (comparison of the standard deviation of fitted versus simulated residuals), and outliers (exact binomial test with approximate expectations). To assess whether spatial dependence influenced model inference, we additionally tested for spatial autocorrelation in residuals using Moran's *I* based on camera coordinates. Moran's *I* values close to zero indicate spatial independence of residuals, suggesting that spatial autocorrelation did not bias model estimates. Marginal means and pairwise contrasts were estimated with the *emmeans* package (Lenth 2025). Separate models were run for each livestock–wild ungulate combination.

## 3 Results

During the study period, we recorded more than 20,000 observations of the focal species, and all species were detected in both seasons (Table 1). Horses and cattle were the most frequently observed species. Cattle encounter rates ranged from 0 to 9.33 encounters/day (mean = 0.10, SD = 0.51), with higher values in summer (mean = 0.12, SD = 0.54) than in winter (mean = 0.06, SD = 0.22). Horse encounter rates ranged from 0 to 2.91 encounters/day (mean = 0.09, SD = 0.23), and were likewise higher in summer (mean = 0.10, SD = 0.24) than in winter (mean = 0.06, SD = 0.12). Dog encounter rates were much lower (mean = 0.003, SD = 0.01), with less pronounced differences between summer (mean = 0.004, SD = 0.01) and winter (mean = 0.002, SD = 0.01). For the two wild ungulate species we observed intermediate encounter rates (roe deer: mean = 0.05, SD = 0.09; wild boar: mean = 0.04, SD = 0.08), with slightly higher values in summer (roe deer: mean = 0.06, SD = 0.09; wild boar: mean = 0.05, SD = 0.08) than in winter (roe deer: mean = 0.04, SD = 0.07; wild boar: mean = 0.04, SD = 0.08). For the Iberian ibex and the Iberian wolf not enough data was available for analysis (winter observations were only 30 and 8, respectively; Table 1).

**TABLE 1:** Mean encounter rates [raw detection numbers] per species and season.

Species		Total	Summer	Winter
Cattle	*Bos taurus*	0.10 [6083]	0.12 [5315]	0.06 [768]
Horse	*Equus caballus*	0.09 [6517]	0.10 [5507]	0.06 [1010]
Dog	*Canis lupus familiaris*	0.003 [289]	0.004 [205]	0.002 [84]
Roe deer	*Capreolus capreolus*	0.05 [4125]	0.06 [3207]	0.04 [918]
Wild boar	*Sus scrofa*	0.04 [3302]	0.05 [2660]	0.04 [642]
Iberian ibex	*Capra pyrenaica*	0.001 [147]	0.001 [117]	0.002 [30]
Iberian wolf	*Canis lupus signatus*	0.002 [145]	0.002 [137]	0.002 [8]
Total number of detections		20,608	17,148	3460

*Note:* Data are pooled across years. The dataset includes 430 camera deployments in summer (2015–2023; mean = 48 per year) and 78 in winter (2020–2023; mean = 26 per year).

### 3.1 Seasonal Activity Patterns of Livestock and Wild Ungulates

**FIGURE 2:**
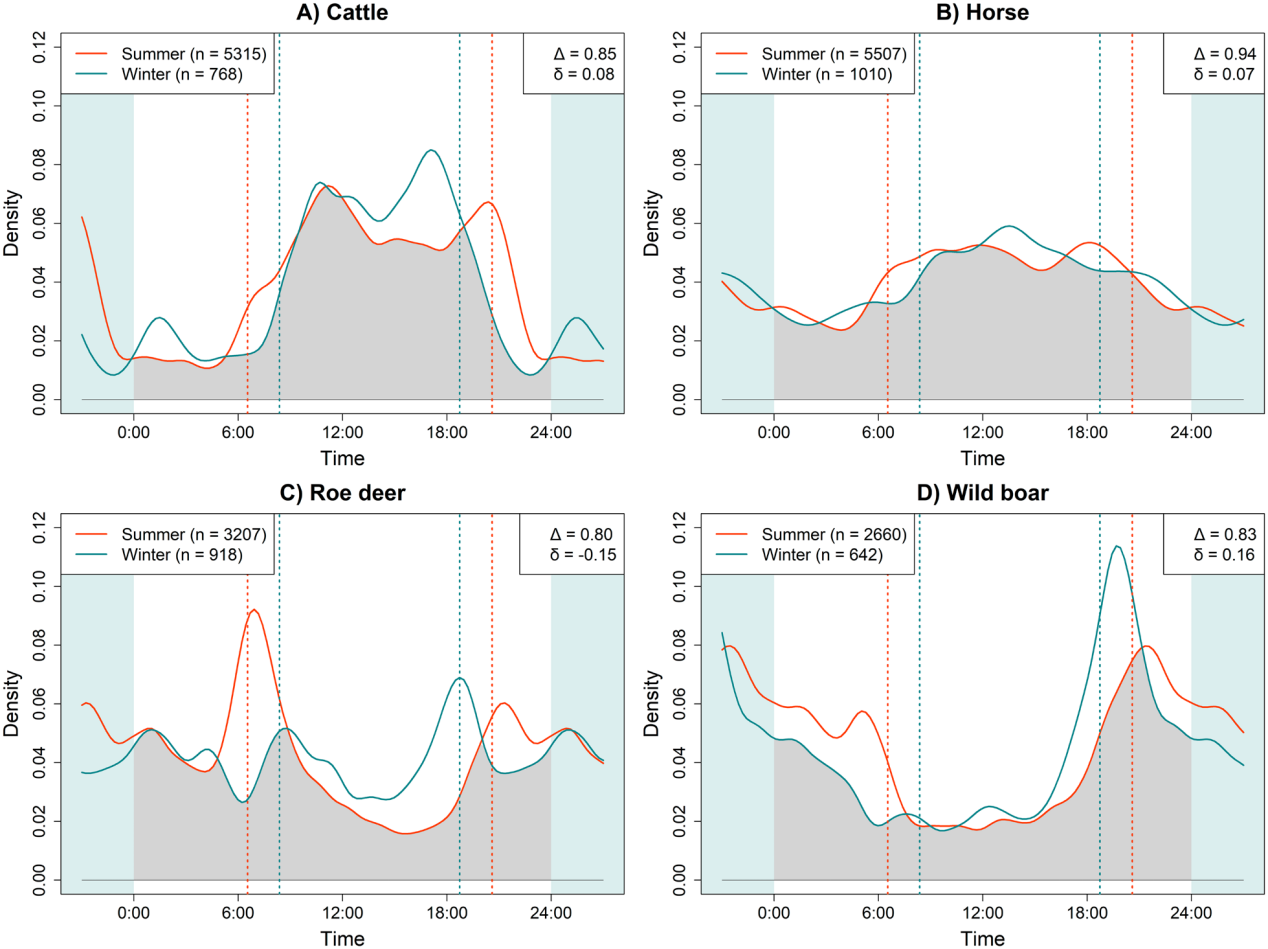
Daily activity patterns of cattle, horses, roe deer, and wild boar in summer (red) and winter (blue), based on kernel density estimates. Vertical dashed lines denote average sunrise and sunset times for each season. Δ indicates the overlap coefficient between seasons, and δ represents the difference in activity levels between seasons.

In both summer and winter, cattle were mainly diurnal, with activity peaking around noon and sunset and most observations occurring between sunrise and sunset (a_day_summer_ = 0.77, a_day_winter_ = 0.80; Figure 2A, Table S1). Nocturnal activity increased slightly in summer, but seasonal differences in patterns (Δ = 0.85, *p* < 0.0001) and levels (δ = 0.08, *p* = 0.025) likely reflect differences in day length between seasons. Herding dogs also showed a clearly diurnal pattern, with activity peaking around noon and slightly higher levels of daytime activity in winter (a_day_summer_ = 0.43, a_day_winter_ = 0.59; a_night_summer_ = 0.08, a_night_winter_ = 0.07, Table S1, Figure S1). Again, differences between activity patterns (Δ = 0.69, *p* < 0.0001) can be attributed to different sunrise times, while activity levels were constant across seasons (δ = −0.01, *p* = 0.850). Horses were active across the full 24‐h cycle, with higher daytime activity but noticeable nighttime movement (a_day_summer_ = 0.92, a_day_winter_ = 0.87; a_night_summer_ = 0.58, a_night_winter_ = 0.58, Table S1). Their activity patterns were largely similar between seasons (Δ = 0.94, *p* = 0.010; δ = 0.07, *p* = 0.146; Figure 2B).

In contrast, roe deer were most active around sunrise and sunset, with slightly higher nocturnal than diurnal activity (a_night_summer_ = 0.55 vs. a_day_summer_ = 0.38; a_night_winter_ = 0.62 vs. a_day_winter_ = 0.58, Table S1). Summer activity peaked around sunrise, whereas in winter it shifted toward sunset, resulting in significant seasonal differences in both activity levels and patterns (Δ = 0.80, *p* < 0.0001; δ = −0.15, *p* = 0.001; Figure 2C). Wild boar activity peaked at sunset in both seasons (Figure 2D) and remained high throughout the night, especially in summer (a_night_summer_ = 0.75 vs. a_day_summer_ = 0.36; a_night_winter_ = 0.44 vs. a_day_winter_ = 0.27, Table S1). In summer, activity was more evenly distributed across the night, whereas in winter it was highest at sunset and then declined, producing significant seasonal differences (Δ = 0.83, *p* < 0.0001; δ = 0.16, *p* < 0.0001; Figure 2D).

### 3.2 Effects of Livestock Abundance on the Temporal Activity of Wild Ungulates

Horses maintained broadly similar daily activity patterns across areas with high and low cattle abundance in both summer and winter (Δ_summer_ = 0.89, Δ_winter_ = 0.92), suggesting limited temporal avoidance of cattle (Figure S2A,B). However, horses showed reduced nocturnal activity where cattle abundance was high, particularly in winter (a_night_high_ = 0.40 vs. a_night_low_ = _0.56), indicating a shift toward more daytime activity in areas with greater cattle presence. The presence of herding dogs was associated with slightly lower horse activity (Figure S2C,D), especially during the day in summer (a_summer_present_ = 0.64 vs. a_summer_absent_ = 0.76), while activity overlap with dogs remained moderate (Δ = 0.62–0.65, Table S2).

**FIGURE 3:**
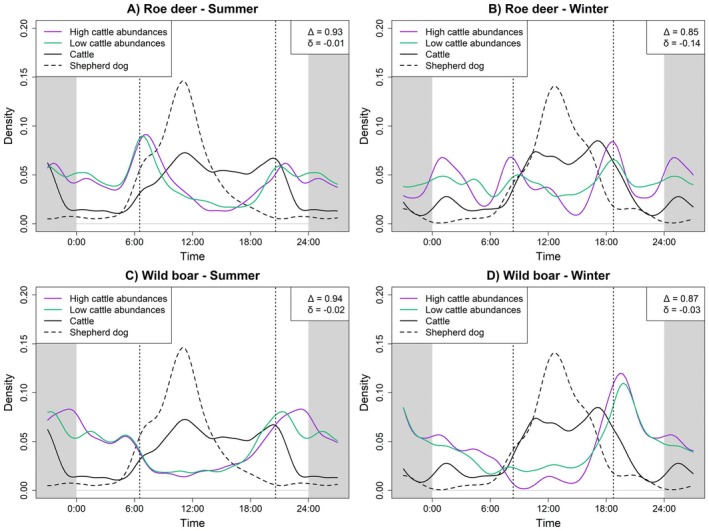
Daily activity patterns of roe deer, and wild boar in summer and winter at locations with high (purple) and low (green) cattle abundance. Cattle (solid black) and herding dog (dashed black) activity patterns are shown for comparison. Vertical dashed lines denote average sunrise and sunset times. Δ indicates overlap in activity patterns between high and low cattle locations, and δ shows the difference in activity levels.

For roe deer, we observed clear seasonal variation in the response to cattle. During summer, there was no clear difference in roe deer activity between high and low cattle abundance sites (Δ = 0.93, *p* = 0.05; δ = −0.01, *p* = 0.86; Figure 3A). In winter, however, roe deer displayed a more diurnal activity pattern at sites with high cattle abundance (Figure 3B, Table S2). At these locations, roe deer activity rose around sunrise, decreased in the afternoon, and peaked again at sunset as cattle activity declined. At low cattle sites, sunrise and sunset peaks were also present but less distinct, with activity more evenly distributed over 24 h and overall higher activity levels (a_total_summer_ = 0.49 vs. a_total_winter_ = 0.63, Table S2). Overall, this resulted in notable, though nonsignificant, differences in both activity patterns and levels (Δ = 0.85, *p* = 0.18; δ = −0.14, *p* = 0.12; Figure 3B). Temporal overlap with cattle was higher in winter (Δ_high_ = 0.66, *p* < 0.0001; Δ_low_ = 0.71, *p* < 0.0001) than in summer (Δ_high_ = 0.63, *p* < 0.0001; Δ_low_ = 0.62, *p* < 0.0001; Table S2), probably due to increased nocturnal activity in summer. Analyses of herding dog activity showed that roe deer activity increased as dog activity declined in summer and winter evenings, especially at high cattle sites, with the drop in dog activity occurring earlier than the drop in cattle activity (Figure 3A,B). After sunrise, roe deer activity decreased at high cattle sites while dog and cattle activity increased, particularly in winter (Figure 3A,B). Overlap analysis with herding dogs showed that while in summer activity levels were largely constant between sites with and without dogs (a_total_present_ = 0.48 vs. a_total_absent_ = 0.46, Table S2), roe deer were more nocturnal at sites with dogs present (a_night_present_ = 0.67 vs. a_night_absent_ = 0.52, Table S2, Figure S3A,B). In winter we observed higher overall activity levels (a_total_present_ = 0.52 vs. a_total_absent_ = 0.65) as well as an increase in diurnal activity when dogs were absent (a_day_present_ = 0.46 vs. a_day_absent_ = 0.71), although roe deer still seemed to decrease activity slightly at peak dog activity times (Figure S3B, Table S2). At sites with dogs present, roe deer showed less overlap with dog activity patterns across seasons (Δ_present_summer_ = 0.45, *p* < 0.0001; Δ_absent_summer_ = 0.53, *p* < 0.0001; Δ_present_winter_ = 0.36, *p* < 0.0001; Δ_absent_winter_ = 0.55, *p* < 0.0001).

**FIGURE 4:**
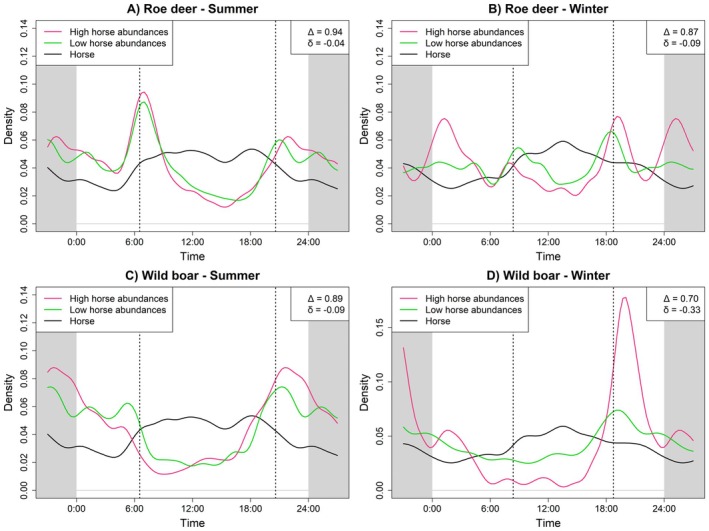
Daily activity patterns of roe deer and wild boar in summer and winter at locations with high (pink) and low (green) horse abundance. Horse (solid black) activity patterns are shown for comparison. Vertical dashed lines denote average sunrise and sunset times. Δ indicates overlap in activity patterns between high and low cattle locations, and δ shows the difference in activity levels.

Similarly, wild boar showed no significant differences in activity patterns or levels between high and low cattle abundance locations in summer (Δ = 0.94, *p* = 0.13; δ = −0.02, *p* = 0.69; Figure 3C). In winter, wild boar activity also did not differ significantly between the two groups (Δ = 0.87, *p* = 0.24; δ = −0.03, *p* = 0.64; Figure 3F), however, during the daytime, wild boar were slightly less active at high cattle sites than at low cattle ones (a_day_low_ = 0.36 vs. a_day_high_ = 0.29, Figure 3D). As with roe deer, we observed an increase of wild boar activity at high cattle locations in the afternoon, which appeared to align with a drop in herding dog activity. In both summer and winter, wild boar activity rose shortly after dog activity declined, while cattle activity remained high for a longer period. Activity pattern overlap with cattle at high cattle abundance locations was higher in summer (Δ_high_ = 0.59, *p* < 0.0001) than in winter (Δ_high_ = 0.50, *p* < 0.0001; Table S2). Again, we observed slightly higher activity levels at sites without herding dog presence in both summer (a_total_present_ = 0.48 vs. a_total_absent_ = 0.55, Table S2) and winter (a_total_present_ = 0.31 vs. a_total_absent_ = 0.46, Table S2), mostly driven by higher levels in diurnal activity (Table S2, Figure S3C,D). Especially in winter, overlap with dog activity times was higher at locations without herding dogs (Δ_present_winter_ = 0.27, *p* < 0.0001; Δ_absent_winter_ = 0.46, *p* < 0.0001). Contrastingly, we observed no strong effect of horses on the temporal activity of wild ungulates in summer (roe deer: Δ = 0.94, *p* = 0.040; δ = −0.04, *p* = 0.377; wild boar: Δ = 0.89, *p* < 0.0001; δ = −0.09, *p* = 0.021; Figure 4A,C), while in winter, there were small but important differences in the activity of both roe deer and wild boar between areas with high and low horse abundance: Roe deer showed slightly higher diurnal activity at locations with low horse abundance (a_day_low_ = 0.65 vs. a_day_high_ = 0.49; Figure 4B, Table S2), resulting in a small difference in activity patterns (Δ = 0.87, *p* = 0.020; δ = −0.09, *p* = 0.299). Temporal overlap with horses was also considerably higher at low horse abundance locations (Δ_high_ = 0.75, *p* < 0.0001; Δ_low_ = 0.86, *p* < 0.0001; Table S2). Similarly, wild boar were less active during the day and showed increased activity around sunset at sites with high horse abundance (a_day_low_ = 0.55 vs. a_day_high_ = 0.20; Figure 4D, Table S2), leading to significant differences in both activity patterns and levels during winter (Δ = 0.70, *p* < 0.0001; δ = −0.33, *p* < 0.0001). Here, the difference in overlap with horse activity patterns depending on horse abundance was even more pronounced (Δ_high_ = 0.52, *p* < 0.0001; Δ_low_ = 0.80, *p* < 0.0001; Table S2).

### 3.3 Seasonal Effects of Livestock Abundance on Wild Ungulate Spatiotemporal Activity

**FIGURE 5:**
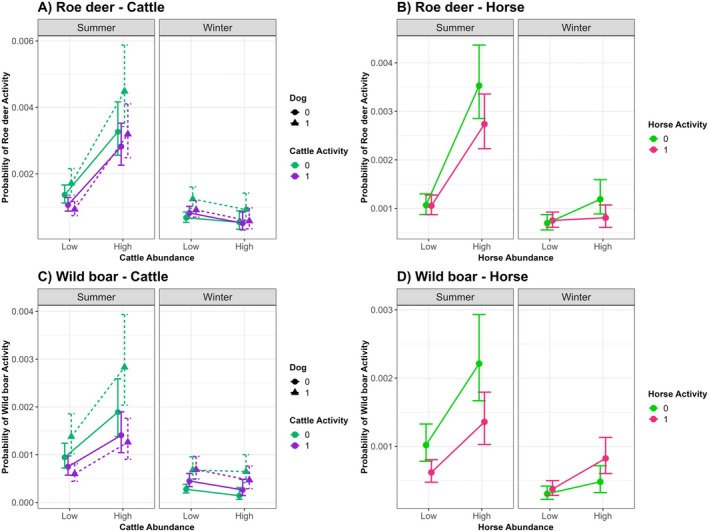
Predicted probabilities of roe deer and wild boar activity across summer and winter seasons, in relation to livestock abundance (Low/High) and livestock activity at the time of detection (1/0), based on binomial generalized linear mixed models (GLMMs). For cattle, dog presence was included as an additional interaction term, denoted by line type (present = dashed, absent = solid). Probabilities reflect the likelihood of species presence within 1‐h time intervals at a given site, with 95% confidence intervals.

The GLMM analysis showed that roe deer occurrence was significantly influenced by cattle abundance, cattle activity, dog presence and season, as well as several interactions. In summer, roe deer occurrence was significantly higher at sites with high cattle abundance (β = 0.87 ± 0.10, *p* < 0.001) and lower during core cattle activity hours (β = −0.25 ± 0.05, *p* < 0.001; Figure 5A). This relationship was more pronounced when dogs were present (Figure 5A), suggesting that herding dogs positively influenced roe deer occurrence (β = 0.23 ± 0.08, *p* = 0.01; Figure 5A).

Interestingly, in winter, this pattern was reversed. Overall roe deer occurrence was lower than in summer (β = −0.70 ± 0.08, *p* < 0.001). Moreover, the relationship between roe deer occurrence and cattle abundance differed significantly between seasons (Cattle × Season: β = −1.09 ± 0.26, *p* < 0.001). As shown in Figure 5A, roe deer occurrence tended to be slightly lower at sites with high cattle abundance during winter, although differences between cattle abundance levels were small and not statistically significant. Seasonal interactions were also detected for cattle activity (β = 0.44 ± 0.11, *p* < 0.001), with a slightly stronger effect of cattle abundance during core cattle activity times, and for dog presence (β = 0.38 ± 0.14, *p* = 0.005) with higher roe deer activity at locations where dogs were present (Figure 5A).

Diagnostic tests indicated no spatial autocorrelation in the residuals (Moran's *I* = −0.12, *p* = 0.999) and dispersion tests based on simulated residuals suggested that the model was not overdispersed (dispersion = 1.37, *p* = 0.112). Although deviations from residual uniformity (Kolmogorov–Smirnov test: D = 0.037, *p* < 0.001) and a slightly elevated number of outliers were detected, inspection of the diagnostic plots did not reveal clear systematic patterns, indicating an overall adequate model fit.

Wild boar showed a similar seasonal pattern. During summer, activity levels were considerably higher at locations with more cattle (β = 0.69 ± 0.11, *p* < 0.001), with activity slightly lower during core cattle activity hours (β = −0.24 ± 0.06, *p* < 0.001; Figure 5B). There was also a positive effect of dog presence (β = 0.38 ± 0.09, *p* < 0.001). Interestingly, however, this effect depended strongly on cattle activity (β = −0.60 ± 0.12, *p* < 0.001), indicating that the positive effect of dogs was weaker during core cattle activity hours (Figure 5B).

Again, we observed a very different pattern in winter. Overall wild boar activity was lower in winter than in summer (β = −1.23 ± 0.11, *p* < 0.001), and cattle abundance tended to have a slightly negative effect, although differences between cattle abundance levels were small and not statistically supported (Figure 5B). The effect of dog presence remained slightly positive in winter.

Model diagnostics for the wild boar‐cattle model yielded results comparable to those observed for roe deer. Residuals showed no evidence of spatial autocorrelation (Moran's *I* = −0.11, *p* = 0.999) and dispersion tests did not indicate overdispersion (dispersion = 1.38, *p* = 0.256). While tests again detected departures from residual uniformity and a slightly higher number of outliers, visual inspection of the residual distributions suggested that these deviations did not reflect major structural issues with the model.

In contrast to cattle, horses showed an almost exclusively positive effect on wild ungulate activity, regardless of species. In summer, both roe deer and wild boar activity increased significantly at sites with high horse abundance (roe deer: β = 1.20 ± 0.08, *p* < 0.001; wild boar: β = 0.78 ± 0.08, *p* < 0.001; Figure 5C,D). For wild boar, activity was lower during core horse activity hours (β = −0.50 ± 0.05, *p* < 0.001; Figure 5D). For roe deer, horse activity alone had no significant effect, but a significant interaction between horse abundance and horse activity (β = −0.24 ± 0.08, *p* = 0.003) indicated that the positive effect of horses was slightly reduced during core horse activity hours (Figure 5C).

In winter, activity levels of both species were lower compared to summer (roe deer: β = −0.43 ± 0.08, *p* < 0.001; wild boar: β = −1.21 ± 0.10, *p* < 0.001; Figure 5C,D). The positive association between ungulate activity and horse abundance was also weaker during winter, particularly for roe deer (Horse × Season: β = −0.67 ± 0.15, *p* < 0.001), although overall patterns remained similar (Figure 5C,D).

Residual diagnostics for the horse models were consistent with those of the cattle models. Neither model showed evidence of spatial autocorrelation (roe deer: Moran's *I* = −0.13, *p* = 0.9999; wild boar: Moran's *I* = −0.11, *p* = 0.9996), and dispersion tests did not indicate overdispersion (roe deer: dispersion = 1.38, *p* = 0.096; wild boar: dispersion = 1.44, *p* = 0.216). Although statistical tests identified deviations from residual uniformity and a modest excess of outliers, diagnostic plots suggested that these deviations were minor and that the fitted models adequately captured the observed patterns.

## 4 Discussion

This study aimed to assess the spatiotemporal activity patterns of wild ungulates in Peneda‐Gerês National Park, Northern Portugal, and to examine how these patterns were influenced by season, as well as the relative abundance and activity of domestic cattle and feral horses, and the presence of herding dogs. We found that in summer, roe deer and wild boar were more active at high cattle abundance sites, suggesting tolerance or even facilitation, with dog presence increasing positive effects for roe deer. However, in winter, both species slightly reduced their activity in response to cattle, indicating spatial and temporal avoidance. In contrast, horse presence was associated with increased wild ungulate activity year‐round, although the effect was much stronger in summer, underscoring the importance of considering both livestock species and seasonal variation in evaluating wildlife‐livestock interactions.

### 4.1 Impact of Season

For all species except horses, activity levels and patterns differed significantly between seasons, with increased nocturnal activity during summer. Cattle remained predominantly diurnal in both summer and winter, consistent with studies from Southern Italy (Gaudiano et al. 2021) and northeastern Spain (Vidal‐Cardos et al. 2025). Horses, by contrast, maintained activity throughout the 24‐h cycle regardless of season, showing only slightly higher diurnal levels, similar to observations from northeast Spain (Vidal‐Cardos et al. 2025). Overall, livestock activity showed little seasonal variation, with minor adjustments likely corresponding to the difference in sunrise and sunset times rather than a shift in activity timing. Wild ungulates, however, displayed more pronounced shifts: roe deer were most active at dawn in summer but shifted their activity peak to dusk in winter, while wild boar showed a comparable pattern, with higher early‐morning activity in summer and stronger post‐sunset peaks in winter. In summer, temperatures rise rapidly during the day and often remain high into the evening, making the early morning the thermally favorable period for activity and leading to reduced activity during hotter hours (Maloney et al. 2005). In contrast, winter temperatures increase throughout the day, such that conditions are most favorable around sunset, likely driving the observed shift in activity toward dusk (Guthmann et al. 2024). Comparable patterns have been reported for wild boar in northern China, where seasonal changes in light, temperature, and precipitation were identified as major drivers of activity shifts (Wang et al. 2024). Similarly, Pagon et al. (2013) reported that roe deer exhibited increased nocturnal activity in summer as a strategy to regulate body temperature. In winter, overall activity appeared lower and more evenly distributed throughout the day, a pattern also documented in northern boreal forest populations (Cederlund 1989). Future studies could improve seasonal resolution by distinguishing all four seasons or focusing on core months (e.g., December–February, June–August) to better capture behavioral phases such as rutting or lactation (Turner 1979), while also addressing potential biases from unequal seasonal sample sizes.

### 4.2 Impact of Livestock

Roe deer and wild boar exhibited broadly similar spatiotemporal responses to livestock presence and activity. In summer, both species showed higher activity probabilities at sites with greater cattle abundance, while in winter activity decreased at high cattle abundance sites. Interestingly, the presence of herding dogs seemed to amplify positive responses to cattle in summer, although this was more evident outside of core cattle activity times, suggesting that ungulate responses were mediated primarily through temporal rather than spatial adjustments. In contrast, horse presence and activity generally promoted wild ungulate activity across seasons, demonstrating a flexible behavioral response to livestock disturbance and interspecific interactions.

Similar patterns have been reported in Mediterranean systems where Gaudiano et al. (2021) reported no temporal partitioning between roe deer and livestock, while Franchetto et al. (2025) found moderate spatial and temporal overlap of roe deer and wild boar with cattle, as well as high temporal overlap with horses during summer. The positive association between wild ungulates and cattle in summer may reflect grazing facilitation. Livestock grazing could be linked to improved food availability and quality through increased vegetation heterogeneity and stimulated regrowth of palatable plants (Gordon 1988; Kie et al. 1991). Activity curves in our study also suggest partial temporal segregation between livestock and wild ungulates, with roe deer and wild boar showing higher activity outside peak cattle activity hours. Such temporal partitioning may allow ungulates to exploit grazed areas while minimizing direct encounters with livestock.

Interestingly, the presence of herding dogs appeared to modify wild ungulate responses to cattle. In summer, positive associations with cattle abundance were more pronounced when dogs were present, although this effect was strongest outside core cattle activity hours. This pattern suggests that dogs influence ungulate behavior primarily through temporal adjustments in activity rather than purely spatial avoidance or attraction. Herding and protection dogs are known to provoke strong behavioral responses in wildlife and can represent an important source of disturbance. For example, Hansen and Smith (1999) documented that dogs frequently pursued or followed moose and roe deer in Norway, often disrupting normal movement and foraging behavior. Similarly, free‐ranging dogs in India have been reported attacking a wide range of native wildlife species, including threatened taxa (Home et al. 2018), and can trigger strong avoidance responses in wild animals (Bhasin et al. 2024). In Peneda‐Gerês National Park, herding dogs are sometimes used to accompany cattle and deter wolf attacks. Consequently, areas where dogs are present may be less frequently used by wolves, potentially reducing perceived predation risk for wild ungulates. Under such conditions, ungulates may tolerate or even prefer areas with livestock and dogs if these areas provide relative refuge from predators. Although our dataset contained too few wolf detections to test this mechanism directly, the observed patterns are consistent with the idea that herding dogs may indirectly influence wildlife behavior by modifying predator presence and the local risk landscape.

In winter, both roe deer and wild boar were significantly less active at high cattle‐abundance sites, suggesting avoidance through spatial and temporal partitioning. Similar seasonal patterns have been observed by Bhasin et al. (2024) in India where overlap between argali and livestock was higher in summer than in winter, as well as by Odadi et al. (2011), who found that in Kenya cattle competed with wild herbivores during the dry season when forage was scarce but benefitted from wildlife grazing during wet‐season periods of high productivity. Comparable responses have been reported in other systems, with wild ungulates avoiding cattle spatially or temporally, but without explicit tests of seasonal effects (Franchetto et al. 2025; Guthmann et al. 2024; Mazzamuto et al. 2025).

This seasonal reversal may thus reflect intensified resource competition or disturbance. When vegetation productivity declines, diet segregation between grazers and browsers often decreases, leading to greater overlap in resource use (Chaikina and Ruckstuhl 2006; Schieltz and Rubenstein 2016). Although roe deer are typically classified as selective browsers specializing on shrubs and woody vegetation, they incorporate more grasses and herbs into their diet when preferred forage becomes scarce (Spitzer et al. 2020; Torres et al. 2011). At the same time, cattle, which are primarily grazers, can increase their consumption of shrubs such as heather and gorse when pasture availability declines (Ferreira et al. 2013; López et al. 2019; Román‐Trufero et al. 2019). These seasonal shifts in feeding strategies may increase dietary overlap between livestock and wildlife and potentially intensify competition for limited plant resources. In addition, cattle in the study area receive supplemental feeding during winter, which may concentrate livestock activity more spatially and increase disturbance or competition at those sites. Under such conditions, wild ungulates may shift toward spatial avoidance of areas with high cattle density.

In contrast to cattle, horses were consistently associated with higher activity of wild ungulates across seasons. Similar positive associations have been reported in other European mountain systems (Franchetto et al. 2025; Mazzamuto et al. 2025; Morera et al. 2023). In the Central Pyrenees, horse densities created moderate vegetation disturbance that increased herbaceous plant availability, improving foraging conditions for wild ungulates (Franchetto et al. 2025). Such facilitative effects are most likely under light to moderate grazing, which can increase plant availability and habitat accessibility, whereas heavy grazing may have detrimental consequences for wild ungulates (Morera et al. 2023). Our findings support the idea that while cattle may act as a source of disturbance, horses exert neutral or facilitative effects on wild ungulates when maintained at sustainable densities that promote habitat heterogeneity (Franchetto et al. 2025).

Nevertheless, responses to livestock can vary widely across ecosystems. In arid and semi‐arid systems, wild ungulates have been shown to avoid dominant species such as feral horses (Hall et al. 2018), likely due to stronger competition for limited resources. Interactions between wild and domestic ungulates therefore depend strongly on ecological context, livestock density, and grazing intensity (Herfindal et al. 2017; Wang et al. 2024). Where both groups coexist, high livestock densities can amplify the effects of niche overlap on wild species, regardless of their competitive strength (Herfindal et al. 2017).

Our results suggest that in temperate mountain systems moderate livestock grazing may facilitate wildlife during productive periods, whereas negative interactions are more likely to emerge during resource‐limited seasons. To meet their energetic demands, wild ungulates likely adjust their spatiotemporal activity to optimize foraging efficiency, reflecting a potential coping strategy (Guthmann et al. 2024; Stewart et al. 2002). To better understand how competition shapes spatial and temporal organization, future research should also integrate data on energy requirements, food availability and preference, as well as habitat carrying capacities.

A key finding of this study is the contrasting seasonal effect of cattle. Such seasonal dynamics are rarely captured in camera trap studies because many analyses focus on summer periods only (Franchetto et al. 2025; Gaudiano et al. 2021; Zuleger et al. 2025) or do not include season in their analyses (Guthmann et al. 2024). As competition for forage intensifies when productivity declines, interactions between wildlife and livestock may shift substantially across seasons even in temperate Mediterranean systems. Our results therefore highlight the need for data from all seasons to accurately assess livestock effects. Restricting analyses to periods of high resource availability can mask important dynamics and lead to incomplete conclusions. Year‐round monitoring is thus essential for developing effective management strategies to mitigate livestock impacts.

## 5 Conclusion and Management Implications

This study investigated how free‐ranging cattle and feral horses affect the temporal activity of wild ungulates in a landscape undergoing agricultural abandonment. Cattle effects varied seasonally, with higher wild ungulate activity at cattle sites in summer but significantly lower activity in winter, suggesting a shift from facilitative interactions during periods of high productivity to potential competition or disturbance when resources are limited. The presence of herding dogs further modified these patterns, with ungulates showing stronger responses to cattle in areas where dogs were present, highlighting the importance of considering livestock‐associated disturbance when evaluating wildlife–livestock interactions. In contrast, horses showed consistently positive effects across seasons, indicating greater compatibility with native ungulates. These results support broader evidence that wild‐domestic ungulate interactions range from facilitation to competition depending on species, resource availability, and associated management practices (Franchetto et al. 2025; Schieltz and Rubenstein 2016). Moderate grazing at low densities can enhance forage quality and habitat heterogeneity, whereas resource‐poor periods or higher stocking rates may promote competition and avoidance. Thus, accounting for species‐ and season‐specific dynamics is crucial, as studies limited to summer may overlook key periods of conflict.

In the context of extensive animal husbandry and agricultural rewilding, our findings suggest that semi‐feral horses can contribute to habitat heterogeneity and coexistence with native ungulates, while the ecological effects of cattle depend strongly on season and management context. In addition to livestock density and grazing intensity, associated factors such as herding dogs may influence wildlife responses by altering disturbance levels or perceived predation risk. Adaptive approaches such as reducing cattle or herding activity in winter, adapting herding practices, including the spatial and temporal use of herding dogs, and maintaining livestock‐free refuges during peak wildlife activity could reduce disturbance and competition. Integrating seasonal and species‐specific management into rewilding frameworks will better align grazing practices with conservation goals while maintaining the ecological functions of pastoral systems.

## Author Contributions

**Melissa C. B. Einsele:** data curation (equal), formal analysis (equal), investigation (equal), methodology (equal), visualization (equal), writing – original draft (equal). **Sandeep Sharma:** conceptualization (equal), methodology (equal), supervision (equal), validation (equal), writing – review and editing (equal). **Matthias Waltert:** supervision (equal), validation (equal), writing – review and editing (equal). **Henrique M. Pereira:** funding acquisition (lead), project administration (lead), resources (lead), supervision (supporting), writing – review and editing (equal). **Annika M. Zuleger:** conceptualization (lead), data curation (equal), formal analysis (equal), investigation (equal), methodology (equal), supervision (equal), visualization (equal), writing – original draft (equal).

## Funding

This work was supported by Deutsche Forschungsgemeinschaft, FZT 118.

## Disclosure

Statement on inclusion: The authors conducted the study in Peneda‐Gerês National Park in coordination with the park authorities. Permissions for fieldwork were obtained, and information on study objectives and outcomes has been provided to local management. While no formal collaboration occurred, we have shared findings and remain open to further engagement in support of conservation and management of the park.

## Conflicts of Interest

The authors declare no conflicts of interest.

## Supporting information


**Figure S1:** Daily activity patterns of herding dogs in summer (red) and winter (blue), based on kernel density estimates. Vertical dashed lines denote average sunrise and sunset times for each season. Δ indicates the overlap coefficient between seasons, and δ represents the difference in activity levels between seasons.
**Figure S2:** (A, B) Daily activity patterns of horses in summer (A) and winter (B) at locations with high (purple) and low (green) cattle abundance. Cattle (solid black) and herding dog (dashed black) activity patterns are shown for comparison. (C, D) Daily activity patterns of horses in summer (C) and winter (D) at locations with herding dogs present (red) and absent (blue). Herding dog (black line) activity patterns are shown for comparison. Vertical dashed lines denote average sunrise and sunset times. Δ indicates overlap in activity patterns between high and low cattle locations, and δ shows the difference in activity levels.
**Figure S3:** Daily activity patterns of roe deer, and wild boar in summer and winter at locations with herding dogs present (red) and absent (blue). Herding dog (black line) activity patterns are shown for comparison. Vertical dashed lines denote average sunrise and sunset times. Δ indicates overlap in activity patterns between high and low cattle locations, and δ shows the difference in activity levels.
**Table S1:** Total (◐), Day (☀) and Night (☾) activity level estimates per species depending on season.
**Table S2:** Total (◐), Day (☀) and Night (☾) activity level estimates per species depending on season and abundance of livestock, as well as activity pattern overlap (Δ) of ungulates with livestock species.

## Data Availability

All data and R scripts for the statistical analysis are available from the Zenodo Repository: https://doi.org/10.5281/zenodo.20209695.
