# Supplementary material for: From Summer Facilitation to Winter Avoidance: Seasonal Shifts in Livestock‐Wild Ungulate Temporal Coexistence
Source: Ecol Evol. 2026 May 24;16(5):e73705. doi: 10.1002/ece3.73705 (PMC13239161; doi:10.1002/ece3.73705)
Supplement: Supplementary file 1 — Figure S1: Daily activity patterns of herding dogs in summer (red) and winter (blue), based on kernel density estimates. Vertical dashed lines denote average sunrise and sunset times for each season. Δ indicates the overlap coefficient between seasons, and δ represents the difference in activity levels between seasons. Figure S2: (A, B) Daily activity patterns of horses in summer (A) and winter (B) at locations with high (purple) and low (green) cattle abundance. Cattle (solid black) and herding dog (dashed black) activity patterns are shown for comparison. (C, D) Daily activity patterns of horses in summer (C) and winter (D) at locations with herding dogs present (red) and absent (blue). Herding dog (black line) activity patterns are shown for comparison. Vertical dashed lines denote average sunrise and sunset times. Δ indicates overlap in activity patterns between high and low cattle locations, and δ shows the difference in activity levels. Figure S3: Daily activity patterns of roe deer, and wild boar in summer and winter at locations with herding dogs present (red) and absent (blue). Herding dog (black line) activity patterns are shown for comparison. Vertical dashed lines denote average sunrise and sunset times. Δ indicates overlap in activity patterns between high and low cattle locations, and δ shows the difference in activity levels. Table S1: Total (◐), Day (☀) and Night (☾) activity level estimates per species depending on season. Table S2: Total (◐), Day (☀) and Night (☾) activity level estimates per species depending on season and abundance of livestock, as well as activity pattern overlap (Δ) of ungulates with livestock species. [file ECE3-16-e73705-s001.docx]

**Supplemental Material**

**Table S1.** Total (◐), Day (☀) and Night (☾) activity level estimates per species depending on season.

| **Species** | | **Season** | **◐** | **☀** | **☾** |
| --- | --- | --- | --- | --- | --- |
| Cattle | *Bos taurus* | Summer | 0.57 | 0.77 | 0.29 |
| Cattle | *Bos taurus* | Winter | 0.49 | 0.80 | 0.25 |
| Horse | *Equus caballus* | Summer | 0.78 | 0.92 | 0.58 |
| Horse | *Equus caballus* | Winter | 0.70 | 0.87 | 0.58 |
| Roe deer | *Capreolus capreolus* | Summer | 0.45 | 0.38 | 0.55 |
| Roe deer | *Capreolus capreolus* | Winter | 0.60 | 0.58 | 0.62 |
| Wild boar | *Sus scrofa* | Summer | 0.52 | 0.36 | 0.75 |
| Wild boar | *Sus scrofa* | Winter | 0.37 | 0.27 | 0.44 |
| Herding dog | *Canis lupus familiaris* | Summer | 0.28 | 0.43 | 0.08 |
| Herding dog | *Canis lupus familiaris* | Winter | 0.30 | 0.59 | 0.07 |

**
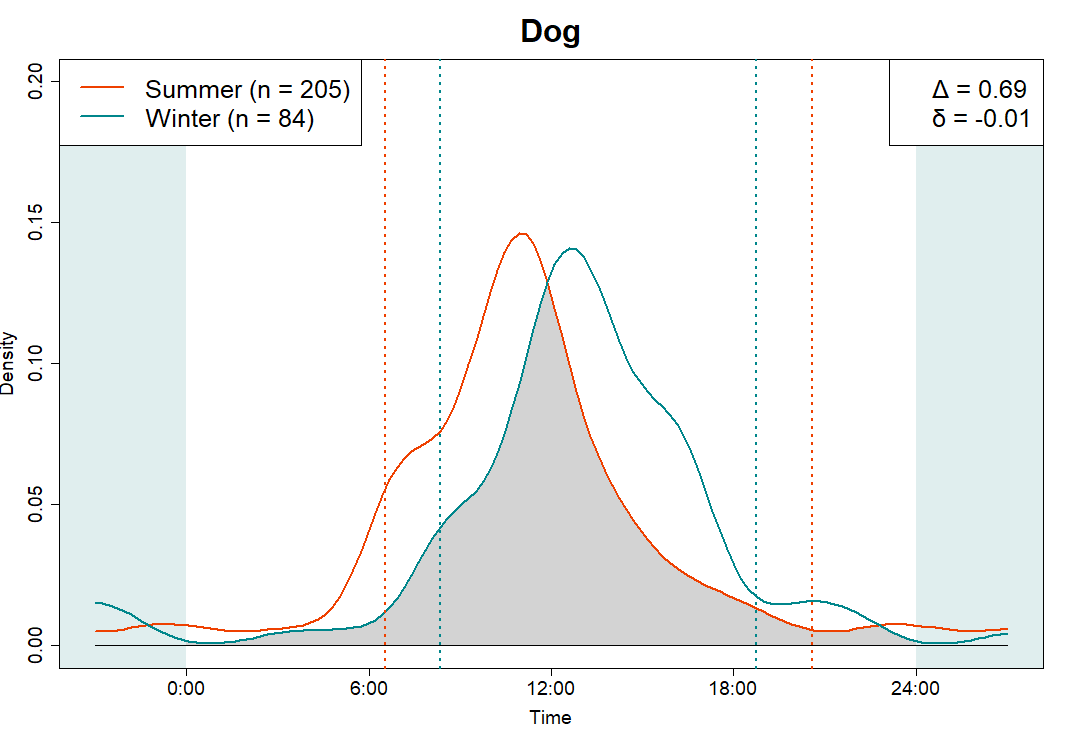
**

**Figure S1.** Daily activity patterns of herding dogs in summer (red) and winter (blue), based on kernel density estimates**.** Vertical dashed lines denote average sunrise and sunset times for each season. Δ indicates the overlap coefficient between seasons, and δ represents the difference in activity levels between seasons.


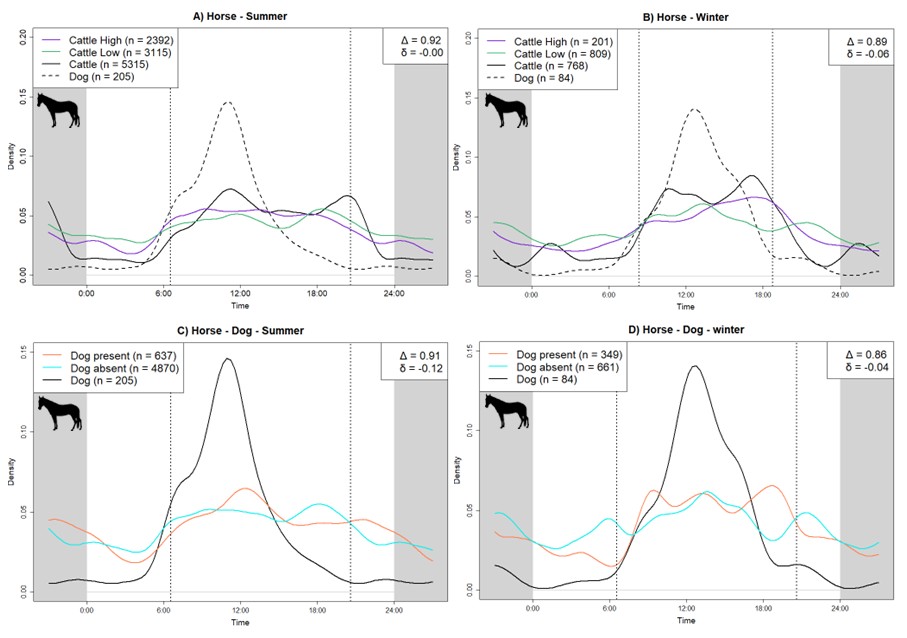


**Figure S2.** A – B) Daily activity patterns of horses in summer (A) and winter (B) at locations with high (purple) and low (green) cattle abundance. Cattle (solid black) and herding dog (dashed black) activity patterns are shown for comparison. C – D) Daily activity patterns of horses in summer (C) and winter(D) at locations with herding dogs present (red) and absent (blue). Herding dog (black line) activity patterns are shown for comparison. Vertical dashed lines denote average sunrise and sunset times. Δ indicates overlap in activity patterns between high and low cattle locations, and δ shows the difference in activity levels.


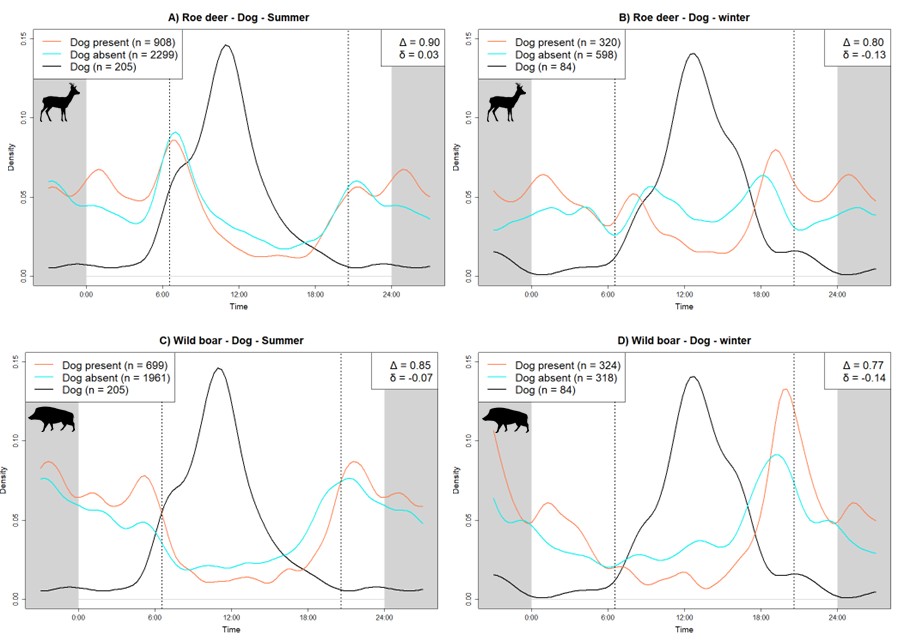


**Figure S3.** Daily activity patterns of roe deer, and wild boar in summer and winter at locations with herding dogs present (red) and absent (blue). Herding dog (black line) activity patterns are shown for comparison. Vertical dashed lines denote average sunrise and sunset times. Δ indicates overlap in activity patterns between high and low cattle locations, and δ shows the difference in activity levels.

**Table S2.** Total (◐), Day (☀) and Night (☾) activity level estimates per species depending on season and abundance of livestock, as well as activity pattern overlap (Δ) of ungulates with livestock species.

| **Species1** | **Species2** | **Season** | **Status** | **◐** | **☀** | **☾** | **Δ** | **P** |
| --- | --- | --- | --- | --- | --- | --- | --- | --- |
| Horse | Cattle | Summer | High | 0.74 | 0.92 | 0.50 | 0.88 | 0.00 |
| Horse | Cattle | Summer | Low | 0.75 | 0.85 | 0.60 | 0.85 | 0.00 |
| Horse | Cattle | Winter | High | 0.63 | 0.79 | 0.40 | 0.87 | 0.00 |
| Horse | Cattle | Winter | Low | 0.68 | 0.77 | 0.56 | 0.79 | 0.00 |
| Horse | Dog | Summer | Present | 0.64 | 0.75 | 0.49 | 0.64 | 0.00 |
| Horse | Dog | Summer | Absent | 0.76 | 0.89 | 0.56 | 0.62 | 0.00 |
| Horse | Dog | Winter | Present | 0.63 | 0.81 | 0.39 | 0.65 | 0.00 |
| Horse | Dog | Winter | Absent | 0.67 | 0.74 | 0.57 | 0.62 | 0.00 |
| Roe deer | Cattle | Summer | High | 0.46 | 0.40 | 0.53 | 0.63 | 0.00 |
| Roe deer | Cattle | Summer | Low | 0.46 | 0.39 | 0.57 | 0.62 | 0.00 |
| Roe deer | Cattle | Winter | High | 0.49 | 0.51 | 0.47 | 0.66 | 0.00 |
| Roe deer | Cattle | Winter | Low | 0.63 | 0.64 | 0.63 | 0.71 | 0.00 |
| Roe deer | Dog | Summer | Present | 0.48 | 0.36 | 0.67 | 0.45 | 0.00 |
| Roe deer | Dog | Summer | Absent | 0.46 | 0.41 | 0.52 | 0.53 | 0.00 |
| Roe deer | Dog | Winter | Present | 0.52 | 0.46 | 0.61 | 0.36 | 0.00 |
| Roe deer | Dog | Winter | Absent | 0.65 | 0.71 | 0.58 | 0.55 | 0.00 |
| Roe deer | Horse | Summer | High | 0.44 | 0.36 | 0.56 | 0.71 | 0.00 |
| Roe deer | Horse | Summer | Low | 0.48 | 0.41 | 0.57 | 0.75 | 0.00 |
| Roe deer | Horse | Winter | High | 0.54 | 0.49 | 0.61 | 0.75 | 0.00 |
| Roe deer | Horse | Winter | Low | 0.63 | 0.65 | 0.60 | 0.86 | 0.00 |
| Wild boar | Cattle | Summer | High | 0.50 | 0.32 | 0.75 | 0.59 | 0.00 |
| Wild boar | Cattle | Summer | Low | 0.52 | 0.36 | 0.73 | 0.62 | 0.00 |
| Wild boar | Cattle | Winter | High | 0.35 | 0.29 | 0.42 | 0.50 | 0.00 |
| Wild boar | Cattle | Winter | Low | 0.38 | 0.36 | 0.40 | 0.61 | 0.00 |
| Wild boar | Dog | Winter | Present | 0.48 | 0.25 | 0.80 | 0.32 | 0.00 |
| Wild boar | Dog | Summer | Absent | 0.55 | 0.41 | 0.74 | 0.38 | 0.00 |
| Wild boar | Dog | Summer | Present | 0.31 | 0.25 | 0.40 | 0.27 | 0.00 |
| Wild boar | Dog | Winter | Absent | 0.46 | 0.49 | 0.41 | 0.46 | 0.00 |
| Wild boar | Horse | Summer | High | 0.47 | 0.32 | 0.69 | 0.66 | 0.00 |
| Wild boar | Horse | Summer | Low | 0.56 | 0.39 | 0.80 | 0.69 | 0.00 |
| Wild boar | Horse | Winter | High | 0.23 | 0.20 | 0.28 | 0.52 | 0.00 |
| Wild boar | Horse | Winter | Low | 0.56 | 0.55 | 0.58 | 0.80 | 0.00 |
